# Patient characteristics and treatment outcomes in marginal zone lymphoma: results of the prospective German MZL registry

**DOI:** 10.1038/s41375-026-02869-7

**Published:** 2026-03-06

**Authors:** Alexander Grunenberg, Birgit Schmelzle, Jacqueline Gökeler, Heike Mund, Andreas Viardot, Jens Dreyhaupt, Gabriele Nagel, Marcel Reiser, Thomas Gabrysiak, Friederike Braulke, Jürgen Dunst, Rudolf Schlag, Tobias Gaska, Volker Runde, Doris Kraemer, Thomas Weber, Lothar Mueller, Patricia Johansson, Carsten Ziske, Georg Hess, Rüdiger Liersch, Christian Buske

**Affiliations:** 1 Department of Internal Medicine III, University Hospital Ulm, Ulm, Germany; 2 Institute of Experimental Cancer Research, CCC Ulm, University Hospital Ulm, Ulm, Germany; 3 Institute of Epidemiology and Medical Biometry, University of Ulm, Ulm, Germany; 4 Praxis Internistischer Onkologie und Hämatologie, PIOH Köln, Köln, Germany; 5 Onkologisches Zentrum, Wolfsburg-Helmstedt, Germany; 6 Comprehensive Cancer Center, Clinic of Hematology and Medical Oncology, University Medical Center, Goettingen, Germany; 7 Department of Radiotherapy, University Hospital Kiel, Kiel, Germany; 8 Hämatologisch-Onkologische Schwerpunktpraxis, Würzburg, Germany; 9 Brüderkrankenhaus St. Josef, Klinik für Hämatologie/Onkologie, Paderborn, Germany; 10 Kath. Karl-Leisner-Klinikum gGmbH Goch, Betriebsstätte Wilhelm-Anton-Hospital, Goch, Germany; 11 Kath. Krankenhaus Hagen gem. GmbH, St.-Josefs-Hospital Hagen, Klinik für Hämatologie und Onkologie, Hagen, Germany; 12 Clinic and Polyclinic for Internal Medicine IV, University Hospital Halle, Halle (Saale), Germany; 13 Praxis Leer, Onkologie UnterEms, Leer, Germany; 14 Department of Haematology, University Hospital Essen, Essen, Germany; 15 St. Josef-Hospital Troisdorf, Practice Network Hematology and Intern. Oncology, Troisdorf, Germany; 16 University of Clinic Mainz, Mainz, Germany; 17 Gemeinschaftspraxis für Hämatologie und Onkologie, Klinik für Innere Medizin, Hämatologie und Onkologie, Clemenshospital Münster, Münster, Germany

**Keywords:** B-cell lymphoma, Diseases

## To the editor

Marginal zone lymphoma (MZL) is an understudied, rare indolent B-cell non-Hodgkin’s lymphoma, though being the second most common indolent lymphoma after follicular lymphoma [1–4]. Although there are national and international guidelines as well as reviews available recommending standard treatments, the overall evidence is weak due to a dearth of larger randomized trials [1, 5, 6]. Of note, the treatment landscape outside clinical trials is largely undefined, and the question remains to which extent national and international recommendations for diagnostics and treatment are transferred into daily clinical management of MZL. Based on this, we initiated a prospective publicly funded registry for MZL. The primary objective of this prospective observational study was to establish a comprehensive overview about clinical characteristics, reporting scope of diagnostic procedures, and real-world treatment patterns for each subtype of MZL patients.

Initiated in 2015, this prospective nationwide non-interventional registry included patients with MZL treated at 49 centers (44 community and 5 academic institutions). For inclusion into the registry, the patients had to be ≥ 18 years of age, and most importantly, needed obligatory confirmation of the diagnosis of MZL by an expert hematopathologist based on the latest WHO classification of lymphoid tumours at the time point of inclusion. Data acquisition was performed online in an open source electronic data capture (EDC) system for clinical studies (LibreClinica 1.0.0, Relia Tec GmbH, 85748 Garching, Germany). Importantly, 100% of the documented patient cases were centrally reviewed and verified by a hematologist to ensure highest data quality. Regular consistency checks and active follow-up concerning diagnostic procedures and treatment were performed to achieve high data completeness and quality. Patients who were on observation, those receiving reclassification, or had missing confirmation of diagnosis by an expert of hematopathology were excluded from further data analysis (Supplementary Fig. 1). In addition, MZL patients diagnosed as CBL-MZ (clonal B-cell lymphocytosis of marginal zone origin) were not included. Among 252 eligible patients with newly diagnosed MZL, follow-up data were actively requested until data cut-off in September 2021, whereas data cut-off analyzing second-line treatment choice was extended to November 2022 (Supplementary Fig. 1). All patients gave written informed consent, and the protocol was approved by the local ethics committee at each participating center. This study complied with the Declaration of Helsinki and its amendments and was done in accordance with Good Clinical Practice guidelines.

Data collection included, among others blastic morphology (also known as large cell MZL variant) and/or proliferation rate, where we considered a cut-off value Ki-67 > 20% as proliferative disease. Risk classification was calculated for each of MZL subtype based on the MALT Lymphoma International Prognostic Index (MALT-IPI) for EMZL (extranodal marginal zone lymphoma) [7], the International Prognostic Index for Follicular Lymphoma (FLIPI) for NMZL (nodal marginal zone lymphoma) [8], and the HPLL Score (named HPLL on the basis of determinant factors) of the SMZL Study Group for SMZL (splenic marginal zone lymphoma) [9]. First-line treatment was subdivided into 6 main categories: anti-infectives, anti-CD20 single agent therapy (anti-CD20), immunochemotherapy (anti-CD20 + CTX), local treatment (radiotherapy (RTX) or surgery, and other treatments (combined treatment regimens and one patient treated by local steroids). In addition, second-line treatment regimens were documented and divided into categories as follows: anti-infectives, anti-CD20 single agent therapy (anti-CD20), immunochemotherapy (anti-CD20 + CTX), CTX (chemotherapy) monotherapy, local treatment (RTX or surgery), autologous stem cell transplantation, and target therapy. Documented response to treatment was assessed by reviewing the physicians' letters in all included patients. Statistical methods are described in the Supplement.

**Fig. 1:**
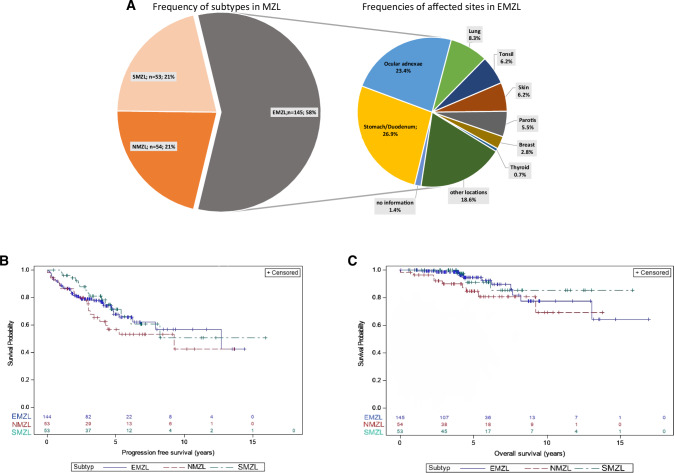
Subtype distribution and treatment outcome. **A** Subtype distribution in MZL; **B** Progression-free survival; **C** Overall survival. MZL Marginal zone lymphoma, EMZL Extranodal marginal zone lymphoma, NMZL Nodal marginal zone lymphoma, SMZL Splenic marginal zone lymphoma.

In total, records of 252 newly diagnosed patients, who were registered within the German MZL registry over a 6-year period, were reviewed. Median follow-up of the patient cohort was 4.5 years (95% CI: 4.2–4.9). 58% (*n* = 145) belonged to the EMZL, 21% (*n* = 54) to the NMZL, and 21% (*n* = 53) to the SMZL subtype, staying in contrast to published retrospective data claiming that the SZML subtype accounts for less than 10% of all MZL [10] (Fig. 1A). Main EMZL manifestations were as follows: upper gastrointestinal tract (27%, *n* = 39), followed by ocular adnexa (23%, *n* = 34) and lung (8%, *n* = 12). Primary cutaneous MZL was reported in 6% (*n* = 9) of patients, which is now considered to be a distinct entity according to the 5th edition of the WHO classification on lymphoid neoplasms, but was analyzed as a subgroup of EMZL in this analysis [3] (Fig. 1A). The median age at diagnosis was 64.5 years (range 18.0-88.0). Every third patient was ≥70 years (35%). Lymphadenopathy was identified in 37% and 42% of all patients with EMZL and SMZL, respectively, demonstrating that also in these subtypes, enlargement of lymph nodes is widespread. Prior oncologic disease at the time of initial diagnosis of MZL was recorded in 17% (*n* = 43) and history of autoimmune disease in 11% (*n* = 27), with no major differences between subgroups. Prognostic scoring according to each MZL risk classification system demonstrated that for EMZL, 55% of the patients belonged to the intermediate/high risk group, 66% for NMZL, and 74% of patients with SMZL. With regard to biological characteristics of disease, either blastic morphology and/or proliferative disease was reported in 12% (*n* = 29/252), with 8% (21/252) of patients showing a Ki-67 positivity of > 20% (Supplementary Table 1). The data set did not classify cases into the category “disseminated MZL” as recently proposed for the MZL-IPI, as this registry was initiated before the introduction of this unifying prognostic score for this lymphoma subtype [11].

Strikingly, evidence of monoclonal gammopathy (52% monoclonal IgM gammopathy, 36% monoclonal IgG gammopathy, 6% IgA gammopathy, 3% light chain, and 3% biclonal gammopathy) was found in 34% (*n* = 36/106) of patients, being detected in 33% of patients with EMZL, 46% in NMZL, and 20% in SMZL. This illustrates that monoclonal gammopathy is a highly recurrent characteristic of MZL, being present in nearly every second NMZL. Every fifth patient did not obtain a bone marrow (BM) biopsy at diagnosis, documenting that in the real-world setting, many patients are managed without any information about BM involvement. Patient characteristics are summarized in Supplementary Table 1.

Data on diagnostic procedures were available for 251 patients, of whom the majority received CT/MRI imaging (89%) at diagnosis (Supplementary Table 1). PET-CT imaging was performed in 12% of the total cohort, with SMZL being the subentity with the lowest rate of PET-CT imaging, reflecting that PET/CT is still not considered standard in this entity. Endoscopic examinations in the form of gastroscopy were performed in 56% of the entire MZL cohort and were documented in 28% of patients with EMZL, of which 27% were of gastric location. Similar findings applied to colonoscopy (EMZL 31%, NMZL 23%, and SMZL 28%) (Supplementary Table 1). Helicobacter pylori positivity was reported in 33% (*n* = 11/33) of the gastric EMZL cohort (Supplementary Table 1).

Median progression-free survival (PFS) for the entire MZL patient cohort, irrespective of subtype, was 12.7 years (95% CI:7.9–NA) with a 5-year PFS of 66% (95% CI: 59–73) and a 5-year OS of 91% (95% CI: 86–95). Median overall survival (OS) was not reached. PFS (*p* = 0.40) and OS (*p* = 0.27) were comparable between the different subtypes. PFS and OS for NMZL were also excellent with a median PFS of 9.2 years (95% CI 3.4-NA) and a 5-year OS of 85% (95% CI 70–92) (Supplementary Tables 2 and 3 and Fig. 1B, C). Overall, progression of disease within 24 months after start of front-line treatment (POD24) frequency, defined as mentioned above, after systemic treatment approaches was low and was observed in only 8% (*n* = 12/142) of patients in this cohort in contrast to observational data describing around 20% of patients with POD24 [12].

Univariate analysis, irrespective of MZL subtype, did not identify any significant factors influencing PFS. Likewise, in multivariate analysis, only elevated lactate dehydrogenase was associated with low impact on PFS, however, without statistical significance (Supplementary Table 4). With regard to potential factors influencing OS, age (HR = 1.10, 95% CI = 1.04–1.15, *p* = 0.0002) was identified as a significant predictor in univariate analysis. In multivariate analysis, independent factors of shorter OS were age (HR = 1.11, 95% CI = 1.04–1.18, *p* = 0.0011) and BM infiltration (HR = 8.95, 95% CI = 2.03–39.40, *p* = 0.0037). However, interpretation of these results should be done with caution as only few deaths occurred, among them only two cases marked as lymphoma -specific deaths.

Paradoxically advanced stage disease was associated with prolonged OS in this exploratory analysis and might be due to statistical coincidence (HR = 0.25, 95% CI = 0.07–0.95, *p* = 0.04) as shown in Supplementary Table 5.

**Fig. 2:**
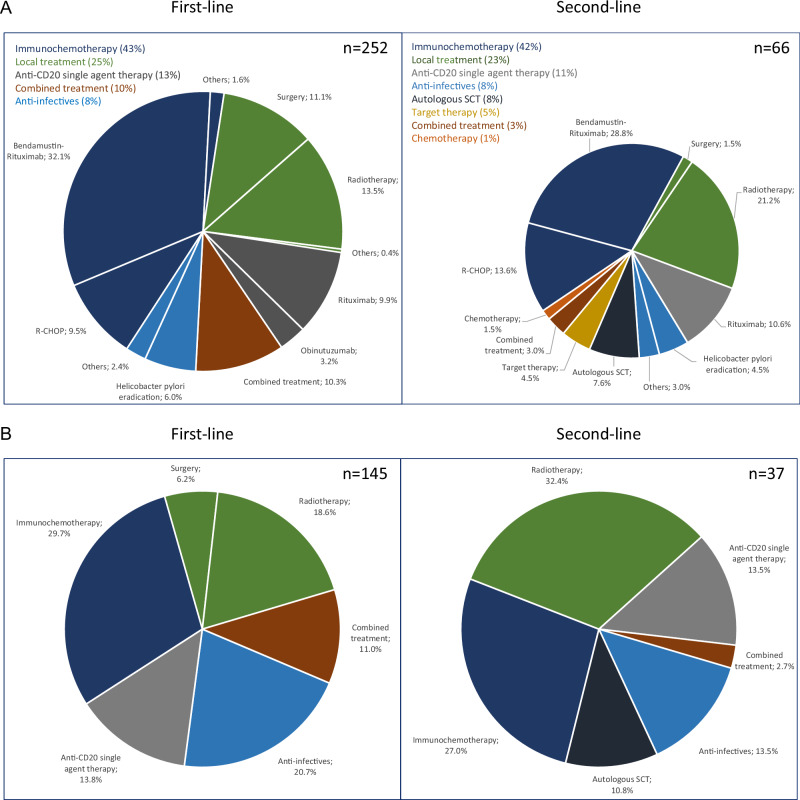

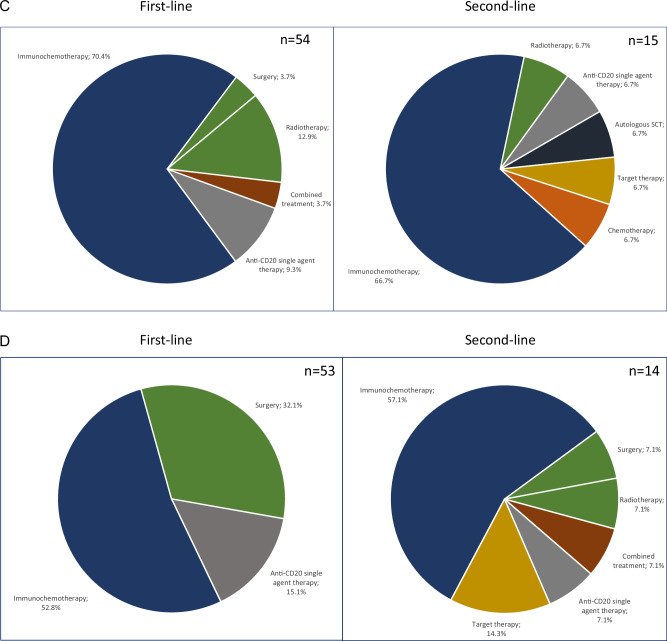
Treatments in MZL. **A** First line and Second line treatment (whole MZL cohort): **B** First line and Second line treatment (EMZL); **C** First line and Second line treatment (NMZL); **D** First line and Second line treatment (SMZL). MZL Marginal zone lymphoma, EMZL Extranodal marginal zone lymphoma, NMZL Nodal marginal zone lymphoma, SMZL Splenic marginal zone lymphoma, SCT Stem cell transplantation.

Median time between diagnosis and start of first-line treatment was 1 month (range 0–64 months). Among first-line treatment of 252 MZL patients, the 3 most common approaches included anti-CD20 + CTX (43%), local treatment (25%), and anti-CD20 single agent therapy (13%). Within the group of patients who received anti-CD20 + CTX, the two most common regimens were rituximab/bendamustine (32%) and R-CHOP (10%). Patients (*n* = 6) receiving single-agent obinutuzumab were participants of the German OLYMP-1 trial (NCT-No 03322865). 8% of the total MZL cohort was treated by antibiotics, all of whom were EMZL patients (Fig. 2A). There were major differences in the use of second-line treatment between the different subgroups: in NMZL, anti-CD20 + CTX dominated, and around two of three patients received this treatment category at relapse. In contrast, only 30% received anti-CD20 + CTX in EMZL as salvage treatment. Of note, every third patient underwent surgery (splenectomy) at relapse in the case of SMZL (Fig. 2B–D). These data demonstrate the large heterogeneity of treatment approaches used to manage MZL in the real-world setting, also indicating that splenectomy is still widely used for relapsed SMZL. Response data of the entire MZL cohort were available for 248 of 252 patients (2 patients lost to follow-up and 2 patients with unknown response status at data cut-off). The overall response rate (ORR) after first line induction was 88.7%. In more detail, categories of response were as follows: complete response (CR) 57.7% (*n* = 143), partial response (PR) 31% (*n* = 77), stable disease (SD) 8.9% (*n* = 22), and progressive disease (PD) 2.4% (*n* = 6) (Supplementary Table 6 and Supplementary Fig. 2). Response data in EMZL were available from 143 (98.6%) with an ORR of 83.2% of patients and a CR rate of 60.1% (*n* = 86) (Supplementary Table 6 and Supplementary Fig. 2). There were high overall response rates after anti-CD20 single agent therapy and anti-CD20 + CTX across the different subtypes, the highest documented in EMZL with 71.4% of cases. In the latter subtype, single agent antibiotic treatment did induce a CR in nearly every 4th patient, and only 14% of the patients showed progression (Supplementary Tables 7–9).

Overall, 22% (*n* = 28/128) of patients who completed induction with either anti-CD20 + CTX (*n* = 21) or with anti-CD20 single agent therapy (*n* = 7; obinutuzumab *n* = 6, rituximab *n* = 1) were assigned to anti-CD20 single agent maintenance. Excluding all patients who received obinutuzumab in the OLYMP-1 trial, which offered maintenance as part of the study protocol, 18% (*n* = 22/122) of patients were assigned to maintenance treatment (36% NMZL, 32% EMZL, and 32% SMZL).

At data cut-off, 66 out of 252 (26%) patients (EMZL *n* = 37, NMZL *n* = 15, SMZL *n* = 14) had received a documented second-line treatment. Median time to start of second-line treatment after end of first-line treatment was 21 months (range 0–95 months). Of the 66 who progressed after front-line treatment and initiated second-line therapy, 43% (*n* = 28) received anti-CD20 + CTX. Rituximab/bendamustine was most commonly used (29%, *n* = 19), followed by R-CHOP (14%, *n* = 9). 11% of patients (*n* = 7) received anti-CD20 single agent therapy, and 23% (*n* = 15) were assigned to local treatment (RTX *n* = 14; surgery *n* = 1). Of note, 8% of patients received autologous stem cell transplantation (*n* = 5) (Fig. 2A). At first relapse, anti-CD20 + CTX was dominating in NMZL, as also seen in first line, indicating that a substantial proportion of patients is re-treated with anti-CD20 + CTX. In SMZL, more than half of the patients are salvaged with anti-CD20 + CTX, whereas in relapsed EMZL RTX, anti-CD20 single agent therapy as well as anti-infectives play a major role beside immunochemotherapy. Distribution of second-line treatment types in each MZL subtype is depicted in Fig. 2B–D. A total of 10% (*n* = 24) mortality was reported during the observation period, with only two cases classified as lymphoma-related death (Supplementary Table 10). This indicates that lymphoma—specific survival might be considerably lower than the overall survival rate in this patient population. However, indication of causes of death might be partly of limited accuracy in registries. In addition, the absolute number of lymphoma—related deaths was low in this data set, which would limit the interpretation of cause-specific survival in this case.

In summary, these data provide a comprehensive and nationwide overview about the frequency of the different MZL subtypes, diagnostics, and treatment choice for MZL in a real-world setting. Of note, all patients received reference pathology, and prospectively collected data were reviewed to 100%, thereby ensuring a very high quality of the data presented. Despite the fact that this registry was collected prospectively, this data analysis is limited by possible selection bias. Nevertheless, it allows valuable insights into the distribution between subtypes with a high proportion of SMZL in our cohort, the existence of a substantial fraction of patients with high Ki-67, and, in particular, monoclonal IgM gammopathy. This registry is still actively recruiting and will continue, having included currently more than 850 patients. Thus, future analyses will allow us to describe potential shifts in diagnostic procedures and treatment patterns in MZL in the upcoming era of target agents and T-cell-directed immunotherapy approaches.

## Supplementary information

Supplemental Material clean

Supplemental Figures

## Data Availability

All data generated or analysed during this study are included in this published article and its supplementary information file.
