# Supplementary material for: Patient characteristics and treatment outcomes in marginal zone lymphoma: results of the prospective German MZL registry
Source: Leukemia. 2026 Mar 6;40(4):845–50. doi: 10.1038/s41375-026-02869-7 (PMC13056515; doi:10.1038/s41375-026-02869-7)
Supplement: Supplementary file 1 — Supplemental Material clean [file 41375_2026_2869_MOESM1_ESM.docx]

**Supplemental Data**

**Statistical analysis**

Progression-free survival (PFS) and overall survival (OS) were investigated using the Kaplan-Meier method and the log-rank test for group comparisons. OS was calculated from the first date of MZL diagnosis to death (=event) or the date of last contact (=censored). PFS was defined as the period of time from start of first line treatment until the occurrence of progression/death from any cause, or until the date of last contact (=censored), if a surviving patient had no documented progression. Death (death from any course) was counted as an event for PFS and OS. Time to first treatment was defined as the time interval of MZL diagnosis to start of front-line therapy. The time between first-line therapy and second-line therapy was calculated from end date of front line to start of second-line treatment. POD24 (progressive disease within 24 months) was defined as the start date of first-line treatment to progressive disease within 24 months. Of note, patients receiving anti-infectives, radiotherapy, and surgery as first-line treatment were excluded for POD24 calculation. Summary statistics are descriptive for demographics, clinical characteristics, and treatment patterns. For continuous data, medians are presented and were dichotomized on the basis of usual thresholds. Categorical data were presented as frequencies and percentages. Due to rounding of decimal places, percentages may not add up to 100%. Univariate and multivariate analysis were performed using the Cox proportional hazards model. For multivariate analysis this was carried out as a “complete case analysis” with the patient data, for which all the information of all variables to be included was available. Before selecting the possible influencing factors, a correlation/collinearity analysis was performed in order not to include strongly interdependent variables in the model at the same time. All risk factors with a p-value <0.20 by univariate analysis were included in the multivariate Cox regression. A stepwise selection was used to define the final model. The results are shown as hazard ratio (HR) and 95% confidence intervals (CI) with p values <0.05 considered as statistically significant. No adjustments were made for multiplicity and the results of significance testing should be considered as hypothesis-generating only. Statistical analyses were done with SAS software, version 9.4. Descriptive analyses were performed by SPSS (Version 28.0.1.0) and MS Excel.

**Suppl Table 1: Characteristics of treatment naïve patients**

| **Characteristic** | **Total cohort (MZL)** | | **EMZL** | | **NMZL** | | **SMZL** | |
| --- | --- | --- | --- | --- | --- | --- | --- | --- |
|  | **N** | **%** | **N** | **%** | **N** | **%** | **N** | **%** |
| Total number | **252** | **100** | **145** | **100** | **54** | **100** | **53** | **100** |
| Age in years, median at time of diagnosis (range) | 64.5 (18-88) |  | 63 (18-83) |  | 66 (36-86) |  | 66 (33-88) |  |
| Sex |  |  |  |  |  |  |  |  |
| Female | 122 | 48 | 67 | 46 | 26 | 48 | 29 | 55 |
| Male | 130 | 52 | 78 | 54 | 28 | 52 | 24 | 45 |
| Stage |  |  |  |  |  |  |  |  |
| I | 86 | 34 | 72 | 50 | 10 | 19 | 4 | 7 |
| II | 35 | 14 | 30 | 21 | 3 | 6 | 2 | 4 |
| III | 39 | 15 | 19 | 13 | 20 | 37 | 0 | 0 |
| IV | 92 | 37 | 24 | 17 | 21 | 39 | 47 | 89 |
| B symptoms | 70 | 28 | 36 | 25 | 13 | 24 | 21 | 40 |
| Lymphadenopathy | 127 | 50 | 53 | 37 | 52 | 96 | 22 | 42 |
| History of oncologic disease | 43 | 17 | 23 | 16 | 12 | 22 | 8 | 15 |
| Autoimmune disease | 27 | 11 | 18 | 12 | 6 | 11 | 3 | 6 |
| Performance Status |  |  |  |  |  |  |  |  |
| ECOG 0 | 166/248 | 66 | 103/141 | 73 | 36/54 | 67 | 27/53 | 51 |
| ECOG 1 | 73/248 | 29 | 35/141 | 25 | 13/54 | 24 | 25/53 | 47 |
| ECOG 2 | 7/248 | 3 | 3/141 | 2 | 3/54 | 6 | 1/53 | 2 |
| ECOG 3 | 2/248 | 1 | 0/141 | 0 | 2/54 | 4 | 0/53 | 0 |
| Risk Score* |  |  |  |  |  |  |  |  |
| Low | 87/228 | 38 | 57/128 | 45 | 17/50 | 34 | 13/50 | 26 |
| Intermediate | 99/228 | 43 | 50/128 | 39 | 19/50 | 38 | 30/50 | 60 |
| High | 42/228 | 18 | 21/128 | 16 | 14/50 | 28 | 7/50 | 14 |
| Laboratory diagnostics |  |  |  |  |  |  |  |  |
| Hemoglobin <10 g/dl | 28/242 | 12 | 8/137 | 6 | 5/53 | 9 | 15/52 | 29 |
| Platelets <100 G/l | 17/245 | 7 | 2/138 | 1 | 3/54 | 6 | 12/53 | 23 |
| Leukocytes >20 G/l | 11/246 | 4 | 0/139 | 0 | 1/54 | 2 | 10/53 | 19 |
| LDH (>LLN) | 49/232 | 21 | 15/127 | 12 | 6/53 | 11 | 28/52 | 54 |
| ß-2 microglobulin (>LLN) | 38/69 | 55 | 16/37 | 43 | 9/17 | 53 | 13/15 | 87 |
| Monoclonal gammopathy | 36/106 | 34 | 19/58 | 33 | 13/28 | 46 | 4/20 | 20 |
| FACS positive (peripheral blood) | 41/86 | 48 | 6/35 | 17 | 9/18 | 50 | 26/33 | 79 |
| BM positive | 85/202 | 42 | 18/106 | 17 | 21/44 | 48 | 46/52 | 88 |
| Imaging methods |  |  |  |  |  |  |  |  |
| CT/MRI | 223/251 | 89 | 126/144 | 88 | 52/54 | 96 | 45/53 | 85 |
| PET-CT | 30/251 | 12 | 21/144 | 15 | 7/54 | 13 | 2/53 | 4 |
| Gastroscopy | 107/251 | 56 | 41/144 | 28 | 20/54 | 37 | 16/53 | 30 |
| Colonoscopy | 71/251 | 28 | 44/144 | 31 | 15/54 | 28 | 12/53 | 23 |
| Ultrasound | 158/251 | 63 | 77/144 | 53 | 36/54 | 67 | 45/53 | 85 |
| Infections |  |  |  |  |  |  |  |  |
| Hepatitis B serology + (n=111) | 10 | 9 | 6/67 | 9 | 2/25 | 8 | 2/19 | 11 |
| Hepatitis C serology + (n=119) | 4 | 3 | 3/67 | 4 | 1/28 | 4 | 0/24 | 0 |
| Helicobacter pylori + (n=32) | 10 | 31 | 10/32 | 31 | NA | NA | NA | NA |
| Biological characteristics |  |  |  |  |  |  |  |  |
| Blastic morphology and/or proliferative variant | 29/252 | 12 | 15/145 | 10 | 13/54 | 24 | 1/53 | 2 |
| Ki-67 % 0-10 | 70/132 | 53 | 46/85 | 54 | 14/35 | 40 | 10/12 | 83 |
| Ki-67 % 11 -20 | 35/132 | 27 | 24/85 | 28 | 10/35 | 29 | 1/12 | 8 |
| Ki-67 % 21 - 30 | 5/132 | 4 | 2/85 | 2 | 3/35 | 9 | 0/12 | 0 |
| Ki-67 % 31 - 40 | 9/132 | 7 | 5/85 | 6 | 4/35 | 11 | 0/12 | 0 |
| Ki-67 % >40 | 13/132 | 10 | 8/85 | 9 | 4/35 | 11 | 1/12 | 8 |

MZL= Marginal zone lymphoma, EMZL=Extranodal marginal zone lymphoma, NMZL=Nodal marginal zone lymphoma, SMZL=Splenic marginal zone lymphoma, ECOG=Eastern Cooperative Oncology Group, LLN=lower limit of normal, FACS= Fluorescence Activating Cell Sorting, CT=Computed tomography, MRI=Magentic Resonance Imaging, PET=Positron emission tomography,*Risk classification was calculated for each of MZL subtype based on the MALT Lymphoma International Prognostic Index (MALT-IPI) for EMZL (extranodal marginal zone lymphoma), the International Prognostic Index for Follicular Lymphoma (FLIPI) for NMZL (nodal marginal zone lymphoma) and the HPLL Score for SMZL. The sum product of the individual risk groups of each score was calculated for the total cohort

**Suppl Table 2: Progression free survival of newly diagnosed MZL**

|  | 2 y | (95% CI) | 5 y | (95% CI) | 10 y | (95% CI) |
| --- | --- | --- | --- | --- | --- | --- |
| Total cohort MZL | 84% | 79-88 | 66% | 58-72 | 52% | 39-62 |
| EMZL | 81% | 73-87 | 68% | 57-77 | 57% | 41-70 |
| NMZL | 82% | 69-90 | 57% | 40-70 | 43% | 20-63 |
| SMZL | 94% | 83-98 | 71% | 54-83 | 51% | 26-71 |

EMZL=Extranodal marginal zone lymphoma, NMZL=Nodal marginal zone lymphoma, SMZL=Splenic marginal zone lymphoma, CI=Confidence interval

**Suppl Table 3: Overall survival of newly diagnosed MZL**

|  | 2 y | (95% CI) | 5 y | (95% CI) | 10 y | (95% CI) |
| --- | --- | --- | --- | --- | --- | --- |
| Total cohort (MZL) | 98% | 96-99 | 91% | 86-94 | 77% | 65-86 |
| EMZL | 99% | 95-99 | 95% | 87-98 | 77% | 58-88 |
| NMZL | 96% | 86-99 | 85% | 70-92 | 69% | 40-86 |
| SMZL | 100% | 100-100 | 91% | 74-97 | 85% | 63-95 |

EMZL=Extranodal marginal zone lymphoma, NMZL=Nodal marginal zone lymphoma, SMZL=Splenic marginal zone lymphoma, CI=Confidence interval

**Suppl Table 4: Univariate and Multivariate Cox Regression Analysis for PFS (total cohort)**

|  | Univariate Cox Proportional Hazard | | | Multivariate Cox Regression Analysis (Final Model) | |
| --- | --- | --- | --- | --- | --- |
| Parameter | n | HR (95% CI) | P Value | HR (95% CI) | P Value |
| Age | 252 | 0.99 (0.98-1.02) | 0.94 |  |  |
| Sex (male vs. female) | 120 vs 130 | 0.93 (0.59-1.45) | 0.74 |  |  |
| Stage (III/IV vs. I/II) | 129 vs 121 | 0.89 (0.57-1.40) | 0.62 |  |  |
| ~~Prognostic~~ Risk Score*  Intermediate vs low | 98 vs 87 | 0.88 (0.52-1.50) | 0.65 |  |  |
| ~~Prognostic~~ Risk Score*  High vs low | 41 vs 87 | 1.05 (0.53-2.08) | 0.90 |  |  |
| ECOG ≥1 vs. 0 | 82 vs 164 | 0.92 (0.57-1.50) | 0.74 |  |  |
| B-Symptoms (yes/no) | 69 vs.181 | 1.25 (0.44-1.26) | 0.73 |  |  |
| Prior oncologic disease (yes/no) | 43 vs. 207 | 1.16 (0.65-2.07) | 0.61 |  |  |
| Autoimmune disorder (yes/no) | 26 vs. 224 | 0.97 (0.46-2.01) | 0.93 |  |  |
| Lymphadenopathy (yes/no) | 126 vs 124 | 1.08 (0.69-1.70) | 0.73 |  |  |
| Bone marrow infiltration (yes/no) | 63 vs 118 | 0.93 (0.52-1.65) | 0.81 |  |  |
| Blastic morphology/proliferation index >20% (yes/no) | 28 vs 221 | 1.34 (0.71-2.56) | 0.36 |  |  |
| LDH >LLN (yes/no) | 48 vs 182 | 1.50 (0.87-2.59) | 0.14 | 0.56 (0.30-1.06) | 0.07 |
| Hemoglobin <10 g/dl (yes/no) | 28 vs 212 | 1.19 (0.59-2.40) | 1.19 |  |  |
| Platelets <100 G/l (yes/no) | 17 vs 226 | 1.24 (0.54-2.86) | 0.61 |  |  |
| Leukocytosis >20 G/l (yes/no) | 9 vs 216 | 1.55 (0.49-4.95) | 0.46 |  |  |
| EMZL vs. SMZL | 144 vs 53 | 1.15 (0.64-2.09) | 0.63 |  |  |
| NZML vs. SMZL | 53 vs 53 | 1.54 (0.79-2.99) | 0.20 |  |  |
| EMZL vs. NMZL | 144 vs 54 | 0.75 (0.44-1.28) | 0.29 |  |  |

ECOG=Eastern Cooperative Oncology Group, LLN= lower limit of normal, EMZL=Extranodal marginal zone lymphoma, NMZL=Nodal marginal zone lymphoma, SMZL=Splenic marginal zone lymphoma, HR=Hazard ratio, PFS=Progression free survival, *Risk classification was calculated for each of MZL subtype based on the MALT Lymphoma International Prognostic Index (MALT-IPI) for EMZL (extranodal marginal zone lymphoma), the International Prognostic Index for Follicular Lymphoma (FLIPI) for NMZL (nodal marginal zone lymphoma) and the HPLL Score for SMZL. The sum product of the individual risk groups of each score was calculated for the total cohort

**Suppl Table 5: Univariate and Multivariate Cox Regression Analysis for OS (total cohort)**

|  | Univariate Cox Proportional Hazard | | | Multivariate Cox Regression Analysis (Final Model) | |
| --- | --- | --- | --- | --- | --- |
| Parameter | n | HR (95% CI) | P Value | HR (95% CI) | P Value |
| Age | 252 | 1.10 (1.04-1.15) | 0.0002 | 1.11 (1.04-1.18) | 0.0011 |
| Sex (male vs. female) | 122 vs 130 | 0.71 (0.31-1.59) | 0.40 |  |  |
| Stage (III/IV vs. I/II) | 131 vs 121 | 1.59 (0.69-3.64) | 0.27 | 0.25 (0.07-0.95) | 0.0418 |
| ~~Prognostic~~ Risk Score*  Intermediate vs low | 99 vs 87 | 2.70 (0.87-8.38) | 0.09 |  |  |
| ~~Prognostic~~ Risk Score*  High vs low | 42 vs 87 | 3.40 (0.93-13.19) | 0.06 |  |  |
| ECOG ≥1 vs. 0 | 82 vs 166 | 1.44 (0.64-3.25) | 0.38 |  |  |
| B-Symptoms (yes/no) | 70 vs.182 | 1.25 (0.53-2.92) | 0.61 | 0.44 (0.15-1.27) | 0.13 |
| Prior oncologic disease (yes/no) | 43 vs. 209 | 1.83 (0.72-4.61) | 0.20 |  |  |
| Autoimmune disorder (yes/no) | 27 vs. 225 | 1.72 (0.59-5.03) | 0.32 |  |  |
| Lymphadnopathy (yes/no) | 127 vs 125 | 2.12 (0.90-4.99) | 0.09 |  |  |
| Bone marrow infiltration (yes/no) | 64 vs 119 | 0.52 (0.17-1.59) | 0.25 | 8.95 (2.03-39.40) | 0.0037 |
| Blastic morphology/proliferation index >20% (yes/no) | 28 vs 223 | 2.36 (0.8-6.35) | 0.08 |  |  |
| LDH > LLN (yes/no) | 49 vs 183 | 1.52 (0,59-3.89) | 0.38 |  |  |
| Hemoglobin <10 g/dl (yes/no) | 28 vs 214 | 0.85 (0.20-3.64) | 0.82 |  |  |
| Platelets <100 G/l (yes/no) | 17 vs 228 | 0.68 (0.09-5.05) | 0.71 |  |  |
| Leukocytosis >20 G/l (yes/no) | 9 vs 218 | 2.01 (0.27-15.12) | 0.50 | 0.14 (0.02-1.30) | 0.0839 |
| EMZL vs. SMZL | 145 vs 53 | 1.16 (0.37-3.66) | 0.80 |  |  |
| NZML vs. SMZL | 54 vs 53 | 2.17 (0.67-7.06) | 0.20 |  |  |
| EMZL vs. NMZL | 145 vs 54 | 0.53 (0.22-1.30) | 0.17 |  |  |

ECOG=Eastern Cooperative Oncology Group, LLN= lower limit of normal, EMZL=Extranodal marginal zone lymphoma, NMZL=Nodal marginal zone lymphoma, SMZL=Splenic marginal zone lymphoma, HR=Hazard ratio, OS=Overall survival, *Risk classification was calculated for each of MZL subtype based on the MALT Lymphoma International Prognostic Index (MALT-IPI) for EMZL (extranodal marginal zone lymphoma), the International Prognostic Index for Follicular Lymphoma (FLIPI) for NMZL (nodal marginal zone lymphoma) and the HPLL Score for SMZL. The sum product of the individual risk groups of each score was calculated for the total cohort

**Suppl Table 6: Treatment response after first-line treatment in MZL (total cohort)**

| Response | | EMZL | NMZL | SMZL | Overall |
| --- | --- | --- | --- | --- | --- |
| CR | n | 86 | 31 | 26 | 143 |
|  | % | 60.1 | 59.6 | 49.1 | 57.7 |
| PR | n | 33 | 18 | 26 | 77 |
|  | % | 23.1 | 34.6 | 49.1 | 31.0 |
| SD | n | 19 | 2 | 1 | 22 |
|  | % | 13.3 | 3.9 | 1.8 | 8.9 |
| PD | n | 5 | 1 | 0 | 6 |
|  | % | 3.5 | 1.9 | 0 | 2.4 |
| Overall | n | 143 | 52 | 53 | 248 |
|  | % | 100 | 100 | 100 | 100 |

EMZL=Extranodal marginal zone lymphoma, NMZL=Nodal marginal zone lymphoma, SMZL=Splenic marginal zone lymphoma, CR=Complete remission, PR=Partial remission, SD=stable disease, PD=Progressive disease

**Suppl Tab 7: Response after first-line treatment in EMZL**

|  | | Anti-infectives | Anti-CD20 | CTX + anti-CD20 | Surgery | RTX | Others | Overall |
| --- | --- | --- | --- | --- | --- | --- | --- | --- |
| CR | n | 7 | 4 | 30 | 9 | 26 | 10 | 86 |
|  | % | 24.1 | 20 | 71.4 | 100 | 96.3 | 62.5 | 60.1 |
| PR | n | 6 | 10 | 11 | 0 | 1 | 5 | 33 |
|  | % | 20.7 | 50 | 26.2 | 0 | 3.7 | 31.3 | 23.1 |
| SD | n | 12 | 6 | 0 | 0 | 0 | 1 | 19 |
|  | % | 41.4 | 30 | 0 | 0 | 0 | 6.2 | 13.3 |
| PD | n | 4 | 0 | 1 | 0 | 0 | 0 | 5 |
|  | % | 13.8 | 0 | 2.4 | 0 | 0 | 0 | 3.5 |
| Overall | n | 29 | 20 | 42 | 9 | 27 | 16 | 143 |
|  | % | 100 | 100 | 100 | 100 | 100 | 100 | 100 |

EMZL=Extranodal marginal zone lymphoma, CR=Complete remission, PR=Partial remission, SD=Stable disease, PD=Progressive disease, CTX=Chemotherapy, RTX=radiotherapy

**Suppl Tab 8: Response after first-line treatment in NMZL**

|  | | Anti-CD20 | CTX + anti-CD20 | Surgery | RTX | Others | Overall |
| --- | --- | --- | --- | --- | --- | --- | --- |
| CR | n | 1 | 22 | 2 | 5 | 1 | 31 |
|  | % | 20 | 61.1 | 100 | 71.4 | 50 | 59.7 |
| PR | n | 4 | 12 | 0 | 1 | 1 | 18 |
|  | % | 80 | 33.3 | 0 | 14.3 | 50 | 34.5 |
| SD | n | 0 | 1 | 0 | 1 | 0 | 2 |
|  | % | 0 | 2.8 | 0 | 14.3 | 0 | 3.8 |
| PD | n | 0 | 1 | 0 | 0 | 0 | 1 |
|  | % | 0 | 2.8 | 0 | 0 | 0 | 2 |
| Overall | n | 5 | 36 | 2 | 7 | 2 | 52 |
|  | % | 100 | 100 | 100 | 100 | 100 | 100 |

NMZL= Nodal marginal zone lymphoma, CR=Complete remission, PR=Partial remission, SD=Stable disease, PD=Progressive disease, CTX=Chemotherapy, RTX=Radiotherapy

**Suppl Table 9: Response after first-line treatment in SMZL**

|  | | Anti-CD20 | CTX + anti-CD20 | Surgery | Overall |
| --- | --- | --- | --- | --- | --- |
| CR | n | 1 | 13 | 12 | 26 |
|  | % | 12.5 | 46.4 | 70.6 | 49.0 |
| PR | n | 7 | 15 | 4 | 26 |
|  | % | 87.5 | 53.6 | 23.5 | 49.0 |
| SD | n | 0 | 0 | 1 | 1 |
|  | % | 0 | 0 | 5.9 | 2 |
| PD | n | 0 | 0 | 0 | 0 |
|  | % | 0 | 0 | 0 | 0 |
| Overall | n | 8 | 28 | 17 | 53 |
|  | % | 100 | 100 | 100 | 100 |

SMZL= Splenic marginal zone lymphoma , CR=Complete remission, PR=Partial remission, SD=Stable disease, PD=Progressive disease, CTX=Chemotherapy

**Suppl Table 10: Cause of death in MZL (total cohort)**

|  | | EMZL | NMZL | SMZL | Overall |
| --- | --- | --- | --- | --- | --- |
| Lymphoma-related deaths | n | 1 | 1 | 0 | 2 |
|  | % | 1 | 2 | 0 | 1 |
| Secondary malignancy | n | 3 | 2 | 1 | 6 |
|  | % | 2 | 4 | 2 | 2 |
| Other related deaths | n | 6 | 5 | 2 | 13 |
|  | % | 4 | 9 | 4 | 5 |
| No information | n | 1 | 1 | 1 | 3 |
|  | % | 1 | 2 | 2 | 1 |
| Overall deaths | n | 11 | 9 | 4 | 24 |
|  | % | 8 | 17 | 7 | 10 |

EMZL=Extranodal marginal zone lymphoma, NMZL=Nodal marginal zone lymphoma, SMZL=Splenic marginal zone lymphoma

**Suppl Figure 1: Definition of patient cohort**

EMZL=Extranodal marginal zone lymphoma, NMZL=Nodal marginal zone lymphoma, SMZL=Splenic marginal zone lymphoma, MZL=Marginal zone lymphoma, CBL-MZ=Clonal B-cell lymphocytosis of marginal zone origin, FL=Follicular lymphoma, DLBCL=Diffuse large B-cell lymphoma, MCL=Mantle cell lymphoma, WM=Waldenstrom macroglobulinemia

**Suppl Figure 2: Treatment response after first-line treatment in MZL**

MZL=Marginal zone lymphoma, EMZL=Extranodal marginal zone lymphoma, NMZL=Nodal marginal zone lymphoma, SMZL=Splenic marginal zone lymphoma, ORR=Overall response rate, CR=Complete Response, PR=Partial response, SD=Stable disease, PD=Progressive disease
