# Supplementary material for: Patient characteristics and treatment outcomes in marginal zone lymphoma: results of the prospective German MZL registry
Source: Leukemia. 2026 Mar 6;40(4):845–50. doi: 10.1038/s41375-026-02869-7 (PMC13056515; doi:10.1038/s41375-026-02869-7)
Supplement: Supplementary file 2 — Supplemental Figures [file 41375_2026_2869_MOESM2_ESM.pptx]

## Slide 1
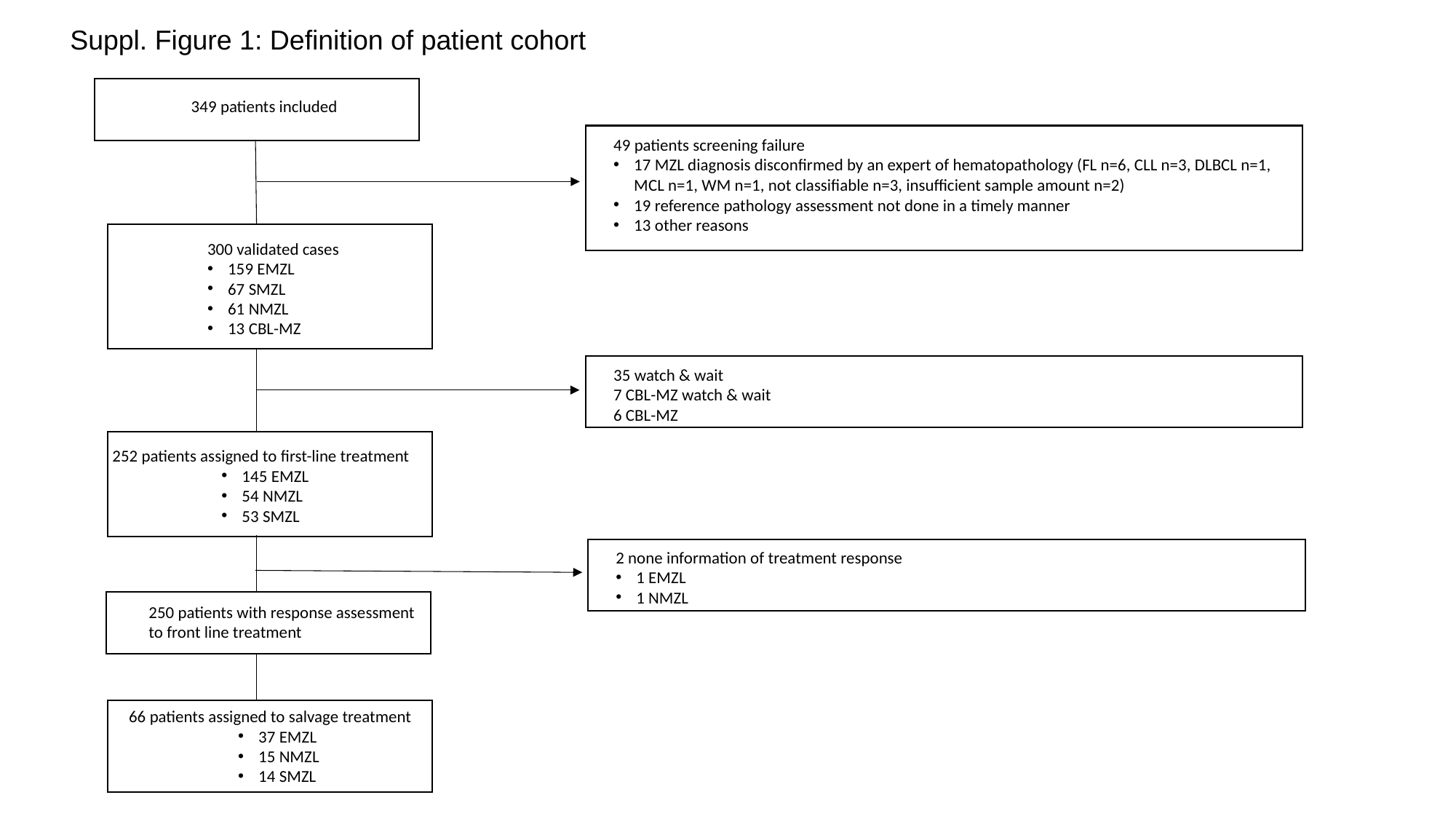

Suppl. Figure 1: Definition of patient cohort
3
349 patients included
3
49 patients screening failure
17 MZL diagnosis disconfirmed by an expert of hematopathology (FL n=6, CLL n=3, DLBCL n=1, MCL n=1, WM n=1, not classifiable n=3, insufficient sample amount n=2)
19 reference pathology assessment not done in a timely manner
13 other reasons
3
300 validated cases
159 EMZL
67 SMZL
61 NMZL
13 CBL-MZ
3
35 watch & wait
7 CBL-MZ watch & wait
6 CBL-MZ
3
252 patients assigned to first-line treatment
145 EMZL
54 NMZL
53 SMZL
3
2 none information of treatment response
1 EMZL
1 NMZL
3
250 patients with response assessment
to front line treatment
66 patients assigned to salvage treatment
37 EMZL
15 NMZL
14 SMZL
3

## Slide 2
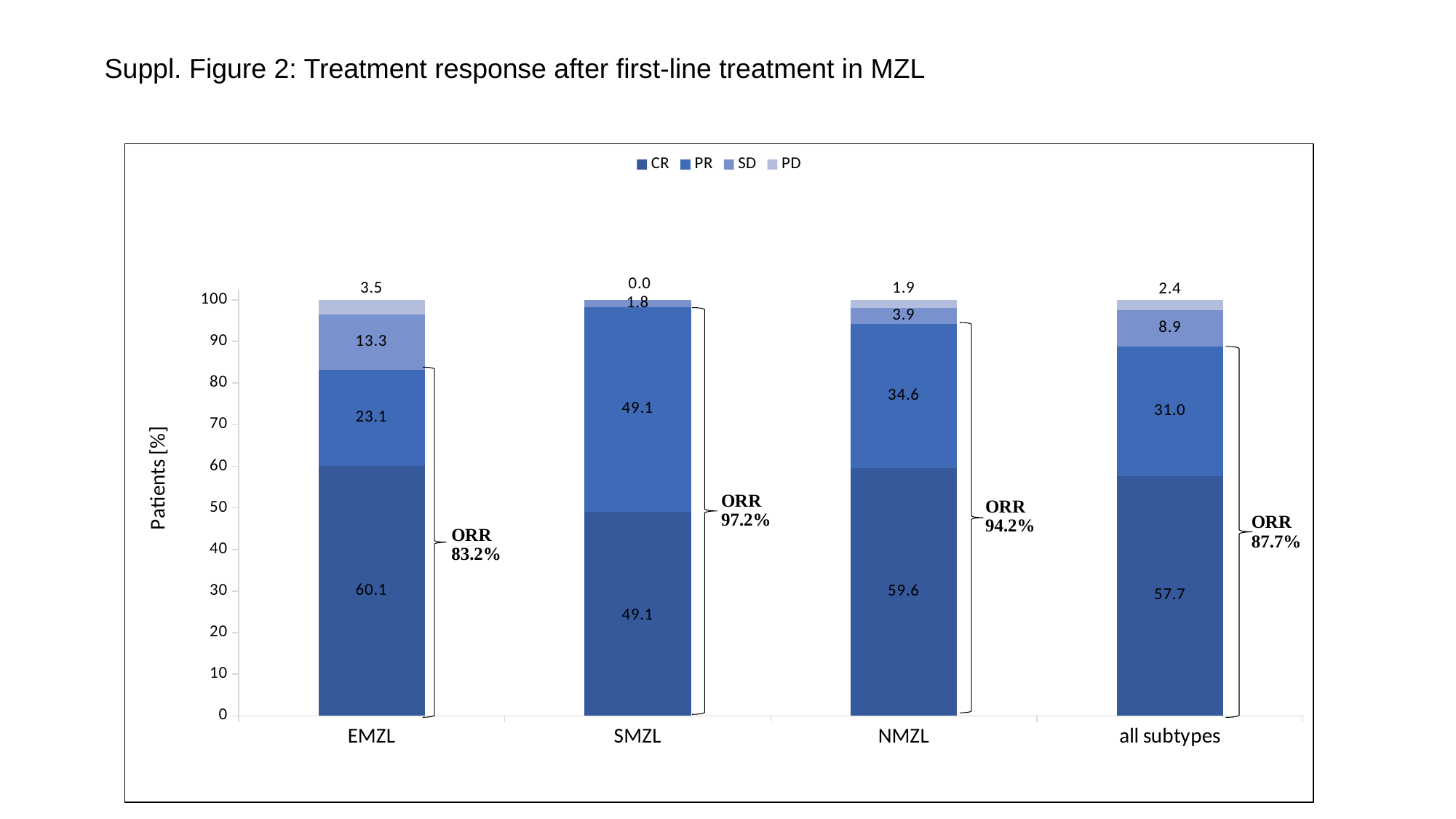

Suppl. Figure 2: Treatment response after first-line treatment in MZL
### Chart
| Category | | | | |
|---|---|---|---|---|
| EMZL | 60.1 | 23.1 | 13.3 | 3.5 |
| SMZL | 49.1 | 49.1 | 1.8 | 0.0 |
| NMZL | 59.6 | 34.6 | 3.9 | 1.9 |
| all subtypes | 57.7 | 31.0 | 8.9 | 2.4 |
